# Unveiling a novel *in-vitro* model of skin inflammaging

**DOI:** 10.3389/fmed.2025.1556680

**Published:** 2025-03-28

**Authors:** Ying Xu, Yue Liu, Junxiang Li, Yao Li, Linlin Xu, Kun Dong, Xiao Lin, Tao Zhang

**Affiliations:** ^1^Better Way (Shanghai) Cosmetics Co. Ltd., Shanghai, China; ^2^AGECODE R&D Center, Yangtze Delta Region Institute of Tsinghua University, Jiaxing, Zhejiang, China; ^3^School of Light Industry Science and Engineering, Beijing Technology and Business University, Beijing, China; ^4^Key Laboratory for Space Biosciences and Biotechnology, School of Life Sciences, Northwestern Polytechnical University, Xi'an, China

**Keywords:** sensitive skin, inflammaging, bakuchiol, *Terminalia chebula* extract, RNA-seq

## Abstract

**Introduction:**

Sensitive skin is characterized by a disrupted skin barrier, making it prone to reacting to external stimuli, including UV exposure, air pollution, and cosmetic allergens. Sensitive skin tends to react with oxidative stress factors that could further lead to inflammation and subsequently result in inflammaging. However, there are almost no existing inflammaging models specifically for sensitive skin, highlighting the need to develop a method for screening anti-inflammaging ingredients and products.

**Methods:**

An *in vitro* macrophage-fibroblast model was established to evaluate the anti-inflammaging effects of the ingredients. The M1 phenotype and aging-associated gene expression were assessed using qPCR to validate the inflammaging model. RNA sequencing was used to further elucidate the inflammaging mechanisms of the two validated ingredients.

**Results and conclusion:**

A novel *in-vitro* model of sensitive skin inflammaging was developed by applying the supernatant of the M1 macrophage culture medium to induce cellular senescence in fibroblast cells, facilitating the screening of anti-inflammaging ingredients. In this model, supramolecular bakuchiol could promote collagen COL1A1 and COL3A3 production and inhibit inflammatory factors by enhancing the transcription of anti-inflammatory genes (*PTX3, ADAM33*, and *PDLIM1*), while *Terminalia chebula* extract inhibits cell senescence by reducing the transcription of MAP4K2 and the accumulation of the inflammatory factor CCL3.

## 1 Introduction

The skin is the largest organ in the human body. As people age, senescent skin cells gradually accumulate and can produce the senescence-associated secretory phenotype (SASP) to affect adjacent cells, resulting in the reduction of skin thickness, regenerative capacity, and barrier effect (1–3). López-Otín et al. proposed 12 hallmarks of aging in 2023 (4). Chronic inflammation, one of the hallmarks of aging, is accompanied by systemic manifestations as well as local pathological phenotypes (5).

Inflammaging skin is a kind of skin aging phenotype resulting from sterile, low-grade chronic inflammation. As aging progresses, inflammation intensifies, generating numerous molecules such as ROS, IL-1, IL-6, IL-8, TNF-α, and MMPs (6, 7), exacerbating skin oxidation and glycation, releasing more free radicals and inflammatory factors while degrading collagen and elastin, thereby accelerating the signs of cellular and skin aging, including wrinkles, sagging, pigmentation, and dullness (8–10).

Sensitive skin, also known as fragile skin or reactive skin, is characterized by a disrupted skin barrier that is often prone to reactions from external stimuli, including UV exposure, air pollution, and cosmetic allergens (11). According to a global questionnaire study, 77.3% of panelists, regardless of gender, reported having sensitive facial skin (12). Compared to normal skin, sensitive skin often experiences chronic inflammation for extended periods, making it more susceptible to inflammaging (13, 14). For those with sensitive skin, anti-inflammaging is a crucial way to prevent skin aging. On one hand, there are only a limited number of *in vitro* models for sensitive skin; the main research studies focus on animal models or clinical studies, which tend to be complex and are not conducive to the rapid screening of cosmetic ingredients (14, 15). On the other hand, there are nearly no models for studying inflammaging in sensitive skin, indicating the need to develop methods for screening anti-inflammaging ingredients and products.

There are several anti-aging ingredients used in cosmetic products for sensitive skin, such as bakuchiol, which is sourced from P*soralea corylifolia Linn* (16, 17). Bakuchiol enhances collagen renewal and refines skin tone and texture, potentially serving as a safer alternative to retinol (18). Supramolecular bakuchiol is composed of bakuchiol, matrine, and vitamin E through non-covalent bonds. Matrine has been proven to possess effective anti-inflammatory properties by significantly inhibiting the production of inflammatory factors such as TNF-α (19). However, further investigation is required to determine whether supramolecular bakuchiol could be applied as a potential agent for anti-skin inflammaging. *Terminalia chebula* extract is another ingredient that has demonstrated notable anti-oxidative, anti-inflammatory, and anti-aging effects (20). Similar to supramolecular bakuchiol, there are limited studies focusing on whether *Terminalia chebula* extract could serve as a potential agent for anti-skin inflammaging due to the lack of an appropriate model.

Horiba et al. showed that M1 macrophages could induce cellular senescence in fibroblast cells (21). Based on this study, we developed a novel *in vitro* model of skin inflammaging by using an M1 macrophage supernatant to induce HSF cell aging. Using this model, we confirmed the anti-inflammaging effects of *Terminalia chebula* extract and supramolecular bakuchiol and aimed to explain the regulatory mechanisms using mRNA-sequence techniques.

## 2 Materials and methods

### 2.1 Experiment materials

The THP-1 cell lines used in this study were obtained from Wuhan Pricella Biotechnology Co., Ltd., while the HSF cell lines were sourced from Newgainbio. *Terminalia chebula* extract (Yingge), matrine, lauric acid (Sigma Aldrich), bakuchiol (Shanghai Youtian Industrial Co., Ltd.), hydroxypinacolone retinoate (Grant Chemical Company), cell culture medium (WISENT, high glucose; Servicebio, RPMI1640 medium), high-quality fetal bovine serum (Gibco), phosphate buffered saline (Basal Media, PBS), trypsin (Basal Media, Trypsin, 0.25%), Cell Counting Kit−8 (Dojindo, CCK-8), cell culture flasks (Thermo, T75), cell culture plates (Corning, 96-well, 6-well, and 12-well), pipettes (Thermo, 5 mL and 15 mL), centrifuge tubes (Corning,15 mL), a disposable cell counting plate (Countstar), a qPCR octuple with an optical flat cap (Abclonal), lipopolysaccharide (Beyotime, LPS), phorbol ester (Beyotime, PMA), interferon-γ (Yufeng Biology, IFN-γ), transforming growth factor β1 (Novoprotein, TGF-β1), RNA extraction kit (Omega), RNA reverse transcription kit (Beyotime), and a 2× Universal SYBR Green Fast qPCR mix (Abclonal) were all utilized in the study.

### 2.2 Experiment equipment

The experiment utilized a variety of equipment, including an adjustable pipette (Eppendorf), an analytical balance (Sartorius), an inverted microscope (Motic), a low-speed centrifuge (HealForce), a refrigerated centrifuge (available instrument), a nucleic acid quantitative instrument (Thermo Fisher), a PCR instrument (Takara), a real-time fluorescence quantitative PCR instrument (Thermo Fisher), a constant temperature incubator (HealForce), an electric pipette (Eppendorf), a cell counter (Countstar), an ultra-clean bench (Thermo), a digital display constant temperature water bath (Lanbao Haibo Biology), a liquid nitrogen tank, a refrigerator (Haier), and a multifunctional microplate reader (PerkinElmer).

### 2.3 Experimental methods

#### 2.3.1 Preparation of supramolecular bakuchiol

Supramolecular bakuchiol was prepared as previously described (22), involving two steps.

Preparation of Mat-LA IL: A specific amount of matrine and lauric acid was dissolved in ethanol and stirred at 30°C for 4 h. The molar ratio of matrine to lauric acid was 1:1. After the reaction was complete, the solvent was removed by vacuum distillation at 60°C, and the resulting matrine lauric acid IL was stored in a vacuum dryer containing phosphorus pentoxide, and it was named Mat-LA IL.

Preparation of supramolecular bakuchiol: A specific quantity of the prepared Mat-LA IL was added to a solution of bakuchiol and stirred in the dark at 30°C for 24 h. The molar ratio of Mat-LA IL to bakuchiol is 1:1. After the reaction was completed, supramolecular bakuchiol was obtained by filtering through a 0.22 μm filter membrane and stored in a light-shielded dryer.

#### 2.3.2 Cell viability assay

The viability of cells was determined using the Cell Counting Kit-8 (Dojindo, CCK-8) according to the manufacturer's instructions (23). Cells were grown in a 96-well plate until they reached 50% to 70% confluency. In the blank control group, 100 μL of culture medium was added to each well. Each well in the sample group received 100 μL of culture medium containing the specified sample concentration. In contrast, the zero group was not inoculated with cells and received only 100 μL of cell culture medium. After adding the samples, the 96-well plate was cultured for 24 ± 2 h. The supernatant was then removed, and the CCK-8 working solution was added and incubated at 37°C for 2 ± 0.5 h in the dark. After incubation, the absorbance was measured at 450 nm. Cell viability was calculated using the following equation according to the manufacturer's instructions.


$$\begin{array}{l} Cell\;viability\;\left(\%\right)=\frac{sample\;group\;OD-zero\;group\;OD}{blank\;group\;OD-zero\;group\;OD}\times 100\% \end{array}$$

#### 2.3.3 *In vitro* model of skin inflammaging

**Figure 1:**
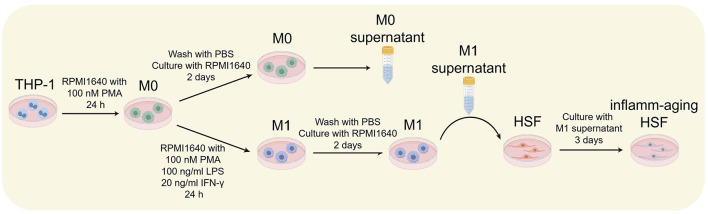
Experimental scheme for the inflammatory aging test, designed using Figdraw (http://www.figdraw.com).

The protocol involved inducing inflammation with M1 macrophages and inducing cellular aging in HSF cells using M1 macrophage supernatant (Figure 1) (21).

##### 2.3.3.1 Induction and identification of M1 macrophages

###### Induction of M1 macrophages:

Induction of M0 Macrophages: THP-1 cells were centrifuged at low speed, resuspended, and counted. A complete RPMI 1640 medium containing 100 nM PMA was prepared. The cells were inoculated in 6-well plates at a density of 1 × 10^6^ cells per well and incubated for 24 h to induce the M0 macrophages.

Induction of M1 Macrophages: RPMI 1640 complete medium containing 100 nM PMA, 100 ng/mL LPS, and 20 ng/mL IFN-γ was prepared. The culture medium of M0 macrophages was removed, and the prepared medium was added to each well. After 24 h, M0 macrophages were induced into M1 macrophages.

###### Identification of M1 macrophages:

M0 group: After the previous step, M0 macrophages were obtained by treating them, and PBS was used for washing. Then, 3 ml of RPMI1640 complete culture medium was added to each well to culture for 2 days, and RNA was extracted.

M1 group: After obtaining M1 macrophages through the previous step, we used PBS for washing. Then, 3 ml of RPMI1640 complete culture medium was added to each well and cultured for 2 days. The supernatant was then collected, and RNA was extracted. To determine whether the expression of CD86, TNF-α, IL-1β, and IL-6 mRNA in M1 macrophages was upregulated compared to M0 macrophages, qRT-PCR was performed. The qRT-PCR primers were 5′-GGAGCGAGATCCCTCCAAAAT-3′ (GAPDH forward) and 5′-GGCTGTTGTCATACTTCTCATGG-3′ (GAPDH reverse); 5′-CCTGCTCATCTATACACGGTTACC-3′ (CD86 forward) and 5′-CGTCGTACAGTTCTGTGACATTATC-3′ (CD86 reverse); 5′-CTCATCTACTCCCAGGTCCTCTTC-3′ (TNF-α forward) and 5′-CGATGCGGCTGATGGTGTG-3′ (TNF-α reverse); 5′-AGGGCTCCTCGGCAAATGTA-3′ (IL-6 forward) and 5′-GAAGGAATGCCCATTAACAACAA-3′ (IL-6 reverse); 5′-TGGCTTATTACAGTGGCAATGAGG-3′ (IL-1β forward) and 5′-AGTGGTGGTCGGAGATTCGTAG-3′ (IL-1β reverse). qRT-PCR assays were conducted under the following cycling conditions: 95°C for 3 min, followed by 40 cycles of 95°C for 5 s and 60°C for 30 s.

##### 2.3.3.2 Induction of inflammaging on HSF cells

HSF cells were seeded in a 12-well plate, achieving a final cell density of 1 × 10^4^ cells per well. The cells were then cultured for 24 h. Various groups were added as indicated and maintained for 3 days. After this period, RNA was extracted, and the expression levels of *p16, p21, COL1A1*, and *MMP-1* genes were detected using qRT-PCR. The qRT-PCR primers used were 5′-GGAGCGAGATCCCTCCAAAAT-3′ (GAPDH forward) and 5′-GGCTGTTGTCATACTTCTCATGG-3′ (GAPDH reverse); 5′-GCCCAACGCACCGAATAGTTAC-3′(p16 forward) and 5′-GCAGCAGCTCCGCCACTC-3′ (p16 reverse); 5′-CCCGTGAGCGATGGAACTTC-3′(p21 forward) and 5′-GCCTGCCTCCTCCCAACTC-3′ (p21 reverse); 5′-CCTGGAAAGAATGGAGATGA-3′(COL1A1 forward) and 5′-ACCATCCAAACCACTGAAAC-3′ (COL1A1 reverse); 5′-ATGAAGCAGCCCAGATGTGGAG-3′(MMP-1 forward) and 5′-TGGTCCACATCTGCTCTTGGCA-3 ′(MMP-1 reverse) . qRT-PCR assays were performed under the following cycling conditions: 95°C for 3 min followed by 40 cycles of 95°C for 5 s and 60°C for 30 s.

#### 2.3.4 RNAseq test for supramolecular bakuchiol and Terminalia chebula extract

Total RNA was extracted using Trizol reagent (Invitrogen, CA, USA) following the manufacturer's protocol. RNA sequencing was conducted by Lianchuan Biotechnology Co., Ltd. (China). Cutadapt software (https://cutadapt.readthedocs.io/en/stable/,version:cutadapt-1.9) was used to eliminate reads that contained adapter contamination (command line: ~cutadapt -a ADAPT1 -A ADAPT2 -o out1.fastq -p out2.fastq in1.fastq in2.fastq -O 5 -m 100).

After removing the low-quality bases and undetermined bases, we used HISAT2 software (https://daehwankimlab.github.io/hisat2/,version:hisat2-2.0.4) to map reads to the genome. The mapped reads for each sample were assembled using StringTie (http://ccb.jhu.edu/software/stringtie/,version:stringtie-1.3.4d.Linux_x86_64) with default parameters (command line: ~stringtie -p 4 -G genome. gtf -o output. gtf -l sample input. bam). Then, all transcriptomes from every sample were merged to reconstruct a comprehensive transcriptome using gffcompare software (http://ccb.jhu.edu/software/stringtie/gffcompare.shtml,version:gffcompare-0.9.8.Linux_x86_64). After generating the final transcriptome, StringTie and ballgown (https://www.bioconductor.org/packages/release/bioc/html/ballgown.html) were used to estimate the expression levels of all transcripts and calculate the expression level for mRNAs by determining FPKM (FPKM = [total_exon_fragments/mapped_reads(millions) × exon_length(kB)]), (command line: ~stringtie -e -B -p 4 -G merged. gtf -o samples. gtf samples. bam).

We then compared the RNA expression levels (FPKM values) across different sample groups using DESeq2 software. Expressed RNA was identified with *P* values < 0.05 and |fold change| >1.5. RNA with fold changes > 1.5 was labeled as upregulated genes (up), while those with fold changes < −1.5 were labeled as downregulated genes (down). Differentially expressed RNAs were visualized using a volcano plot, and the selected differentially expressed RNAs were analyzed with a heatmap. Gene ontology (GO) (http://www.geneontology.org) and Kyoto Encyclopedia of Genes and Genomes (KEGG) (http://www.genome.jp/kegg/) analyses were conducted using bioinformatics, with *p* < 0.05 considered significant, and plotted via https://www.bioinformatics.com.cn, an online platform for data analysis and visualization (24).

### 2.4 Statistical analysis

All experiments were conducted with a minimum of three biological replicates. All experimental data were statistically analyzed using GraphPad Prism 9 software. An unpaired Student's *t*-test was used to assess whether there was a significant difference between the two groups. *P*-values < 0.05 were considered statistically significant.

## 3 Results

### 3.1 Induction and identification of M1 macrophages

**Figure 2:**
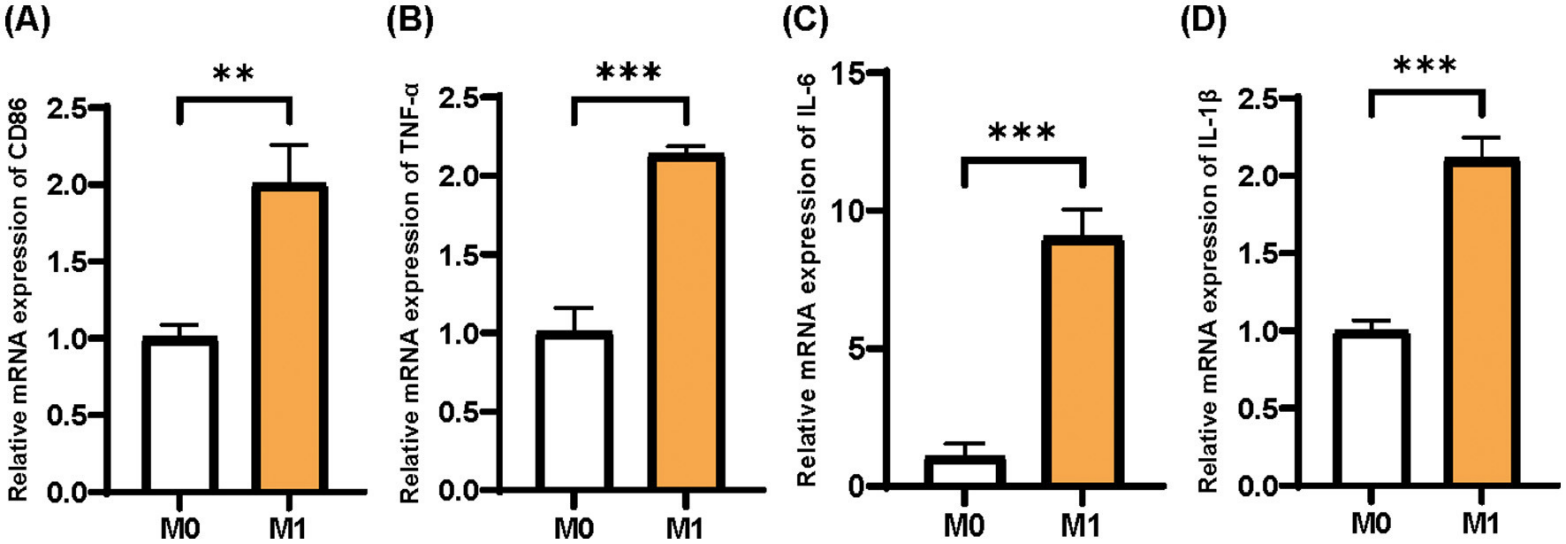
Identification of M0 and M1 phenotypes of THP-1 cells. **(A)** CD86; **(B)** TNF-α; **(C)** IL-6; **(D)** IL-1β. Mean ± SD, *n* = 3.

According to the previous study, CD86, TNF-α, IL-1β, and IL-6 were found to be important inflammatory markers for M1 macrophages induced by THP-1 cells (21). Based on the qPCR test, the mRNA expressions of CD86, TNF-α, IL-1β, and IL-6 in M1 macrophages were significantly higher than those in M0 macrophages, indicating that the induction of M1 macrophages was successful (Figure 2).

### 3.2 Induction of inflammaging on HSF cells

**Figure 3:**
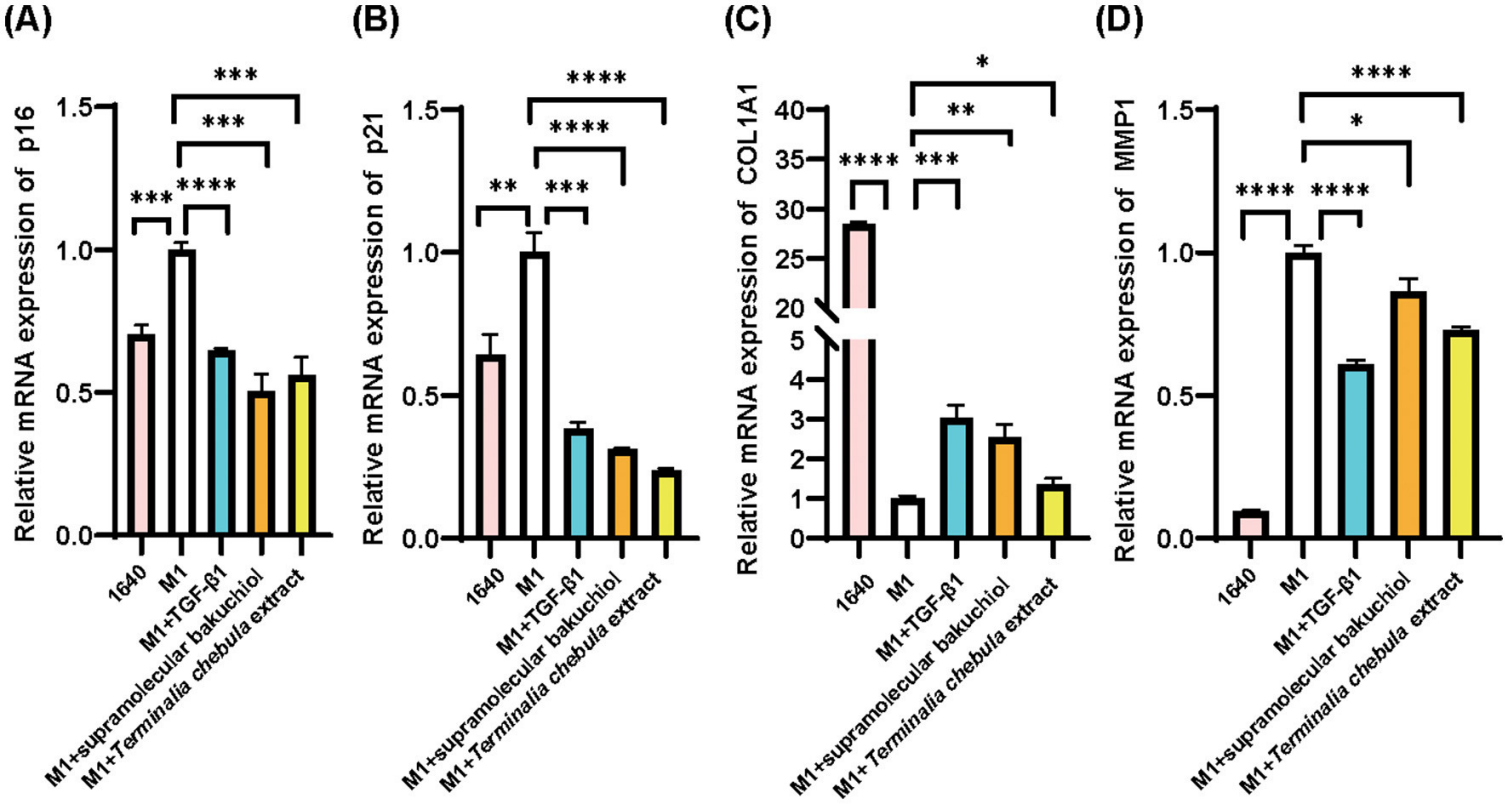
HSF cell inflammatory senescence assay for the results of the anti-inflammatory senescence test of the samples: 1640: RPMI1640 medium, M1: M1 macrophage supernatant, sample group: M1 macrophage supernatant plus each sample (TGF-β, supramolecular bakuchiol, and *Terminalia chebula* extract); **(A)** p16; **(B)** p21; **(C)** Col-1; **(D)** MMP-1. Mean ± SD, *n* = 3.

We collected the culture medium of M1 macrophages and used the supernatant to culture HSF cells. After 3 d, we detected the cellular senescence indicators p16 and p21, along with skin aging-related indicators MMP-1 and Col-1, via qPCR. Compared to the blank control 1,640 group, the expression levels of p16, p21, and MMP-1 mRNA in the negative control M1 group were significantly upregulated, while the expression of Col-1 was significantly downregulated. Additionally, the positive control TGF-β1 group significantly downregulated the expression of p16, p21, and MMP-1, while upregulating the expression of Col-1 (Figure 3). This set of results confirmed the successful induction of the inflammaging process model.

### 3.3 Model validation by using supramolecular bakuchiol and *Terminalia chebula* extract

**Figure 4:**
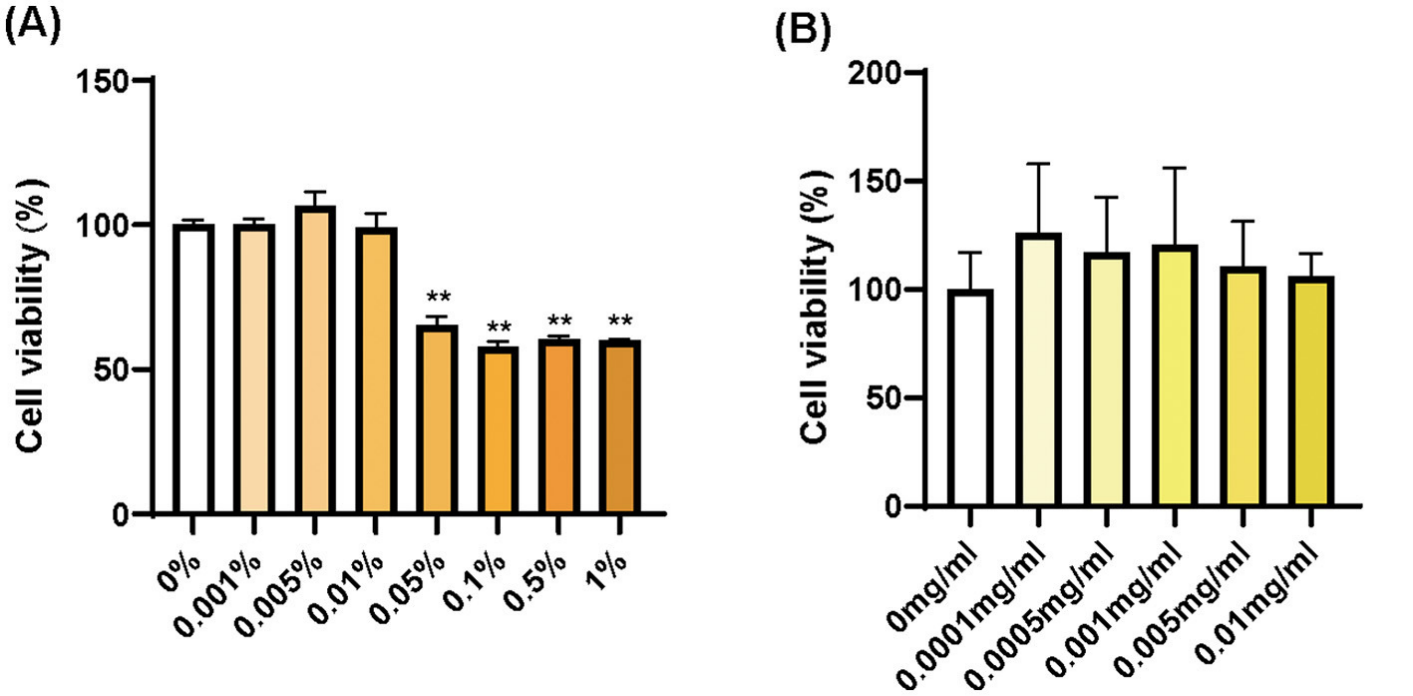
Cell viability in HSF cells for **(A)** supramolecular bakuchiol and **(B)** *Terminalia chebula* extract. Mean ± SD in **(A)**, *n* = 3; in **(B)**, *n* = 4.

To validate the inflammaging models, we chose two anti-aging ingredients and identified the safe concentration range of supramolecular bakuchiol and *Terminalia chebula* extract, which we are interested in, on HSF cells. Based on the cell viability assay, supramolecular bakuchiol shows no cytotoxicity in the concentration range of 0% to 0.01% (the concentration of bakuchiol is 0 to 0.1 mg/mL), with cell viability exceeding 90% (Figure 4A). *Terminalia chebula* extract exhibits no cytotoxicity in the concentration range of 0 to 0.01 mg/mL (Figure 4B).

Based on the cell viability test, we added supramolecular bakuchiol at a concentration of 0.001% (equivalent to 0.01 mg/mL), and *Terminalia chebula* extract at 0.003 mg/mL to the culture medium of HSF cells in our inflammaging model. Both the anti-aging ingredient supramolecular bakuchiol and the anti-inflammatory ingredient *Terminalia chebula* extract significantly downregulated the expression of p16, p21, and MMP-1 while upregulating the expression of Col-1. This indicates that both ingredients could reverse inflammaging in HSF cells, making them suitable for further application in cosmetic formulations aimed at anti-inflammaging properties (Figure 3).

### 3.4 RNAseq test for supramolecular bakuchiol group

To further elaborate on the difference in the inflammaging mechanism between supramolecular bakuchiol and *Terminalia chebula* extract, we extracted total RNA from the HSF cells collected in the inflammaging model. We then performed RNA sequencing and compared the RNA expression levels (FPKM value) of the different sample groups. We screened gene RNA with *P* values < 0.05 and |fold change| >1.5. RNA with a fold change >1.5 was labeled as upregulated genes (up), and those with a fold change < −1.5 were labeled as downregulated genes (down).

**Figure 5:**
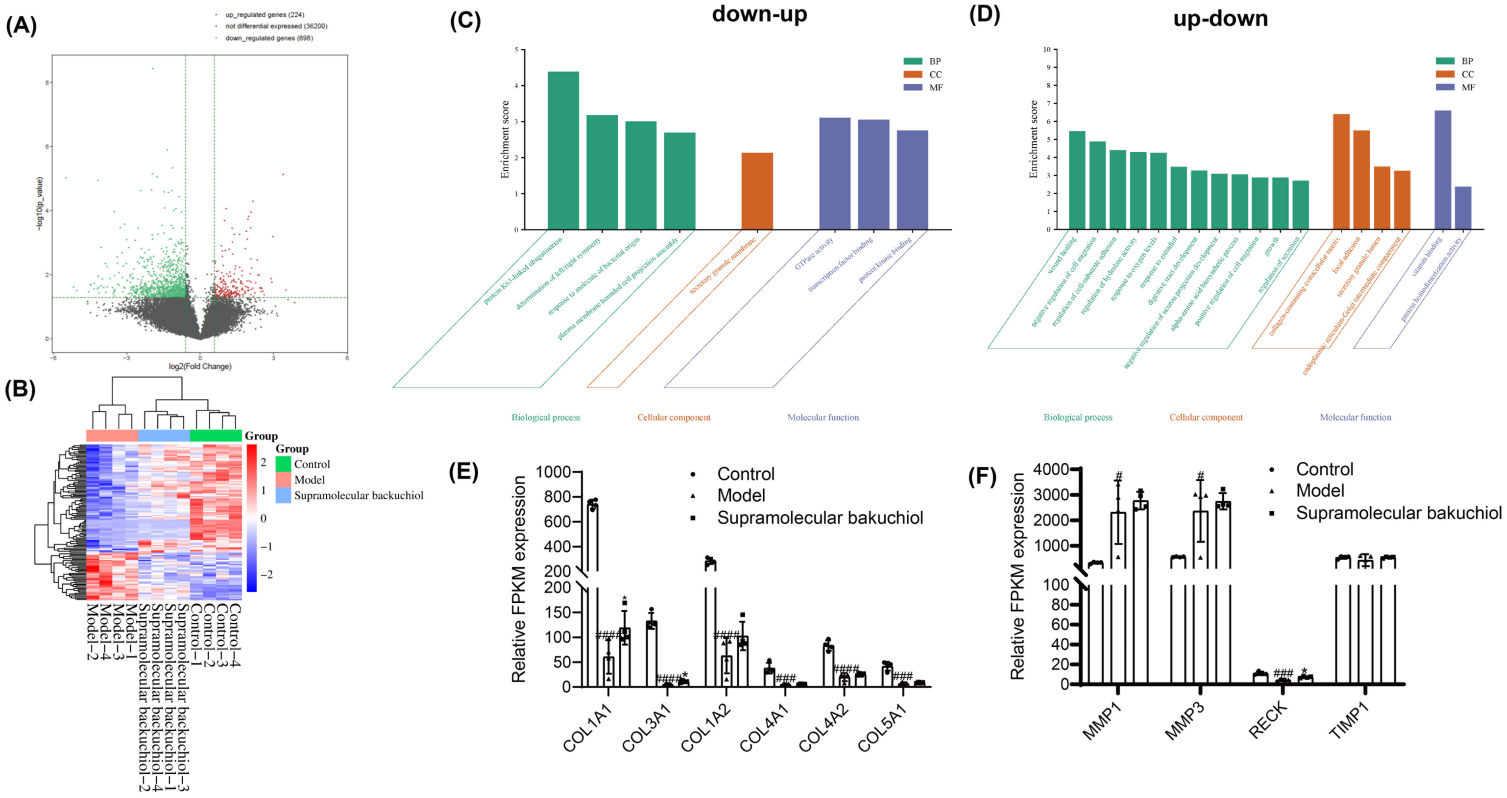
**(A)** A volcano plot comparing the inflammaging group to the supramolecular bakuchiol group for upregulated, non-differentially expressed, and downregulated genes in the transcriptomic profile of inflammatory senescence. **(B)** Cluster analysis of supramolecular bakuchiol focusing on up-down and down-up genes in the transcriptomic profile of inflammatory senescence (Control: 1640: HSF cells treated with RPMI1640 complete medium; Model: HSF cells treated with M1 THP-1 cell medium; Supramolecular bakuchiol group: HSF cells treated with M1 THP-1 cell culture medium and supramolecular bakuchiol; Up-down: Compared to the control group, gene expression in the inflammaging group was upregulated, whereas it was downregulated compared to the supramolecular bakuchiol group; Down-up: Gene expression in the inflammaging group was downregulated relative to the control group and upregulated compared to the supramolecular bakuchiol group; color indicates the level of gene expression, with the red color showing higher expression and blue colors indicating lower expression). **(C, D)** Functional cluster analysis of supramolecular bakuchiol (Up-down: Compared to the control group, gene expression in the inflammaging group was upregulated, whereas it was downregulated compared to the supramolecular bakuchiol group; Down-up: Gene expression in the inflammaging group was downregulated relative to the control group and upregulated compared to the supramolecular bakuchiol group). **(E)** Effect of supramolecular bakuchiol on ECM-related genes. **(F)** Effect of supramolecular bakuchiol on the expression of anti-inflammatory-related genes.

According to the volcano plot, compared to the inflammaging group, the supramolecular bakuchiol group previously upregulated 224 genes and downregulated 898 genes after sample treatment (Figure 5A). We then focused on the upregulated and downregulated genes from the supramolecular bakuchiol group. A total of 136 significantly differentially expressed genes were identified in the control group, the inflammaging group, and the supramolecular bakuchiol group (*p* < 0.05). Compared to the control group, the inflammaging group previously upregulated 38 genes and subsequently downregulated (up-down) after sample treatment. Meanwhile, 86 genes were previously downregulated in the inflammaging group and subsequently upregulated (down-up) after sample treatment. The cluster heat map illustrates the changes in upregulated and downregulated gene expression levels in these three groups (Figure 5B).

The functions of the up-down and down-up related genes were analyzed using the GO database and were annotated with GO terms (Figures 5C, D). The results showed that up-down-related genes mainly affected the cellular molecular function of GTPase activity and the biological process of neuronal projection development. Down-up genes significantly impacted various biological processes, including wound healing, regulation of hydrolase activity, response to oxygen levels, negative regulation of cell migration, muscle structure development, extracellular matrix (ECM) biosynthesis, and alpha-amino acid biosynthesis, among others.

In this experiment, it was found that after treatment with the M1 type THP-1 cell culture medium, the expression of collagen synthesis-related genes, including *COL1A1, COL1A2, COL3A1, COL4A1, COL4A2*, and *COL5A1*, was significantly decreased in HSF cells (25). Additionally, the expression of *COL1A1* and *COL3A3* genes significantly increased following the treatment with supramolecular bakuchiol (Figure 5E). In addition, supramolecular bakuchiol significantly promotes the anti-inflammatory genes *PTX3* (26, 27), *ADAM33* (28, 29), and *PDLIM1* (30), thereby mitigating the impact of the inflammatory environment on HSF cells (Figure 5F). Consequently, supramolecular bakuchiol inhibits inflammaging primarily by promoting collagen production and reducing inflammatory factors.

### 3.5 RNAseq test for *Terminalia chebula* extract group

**Figure 6:**
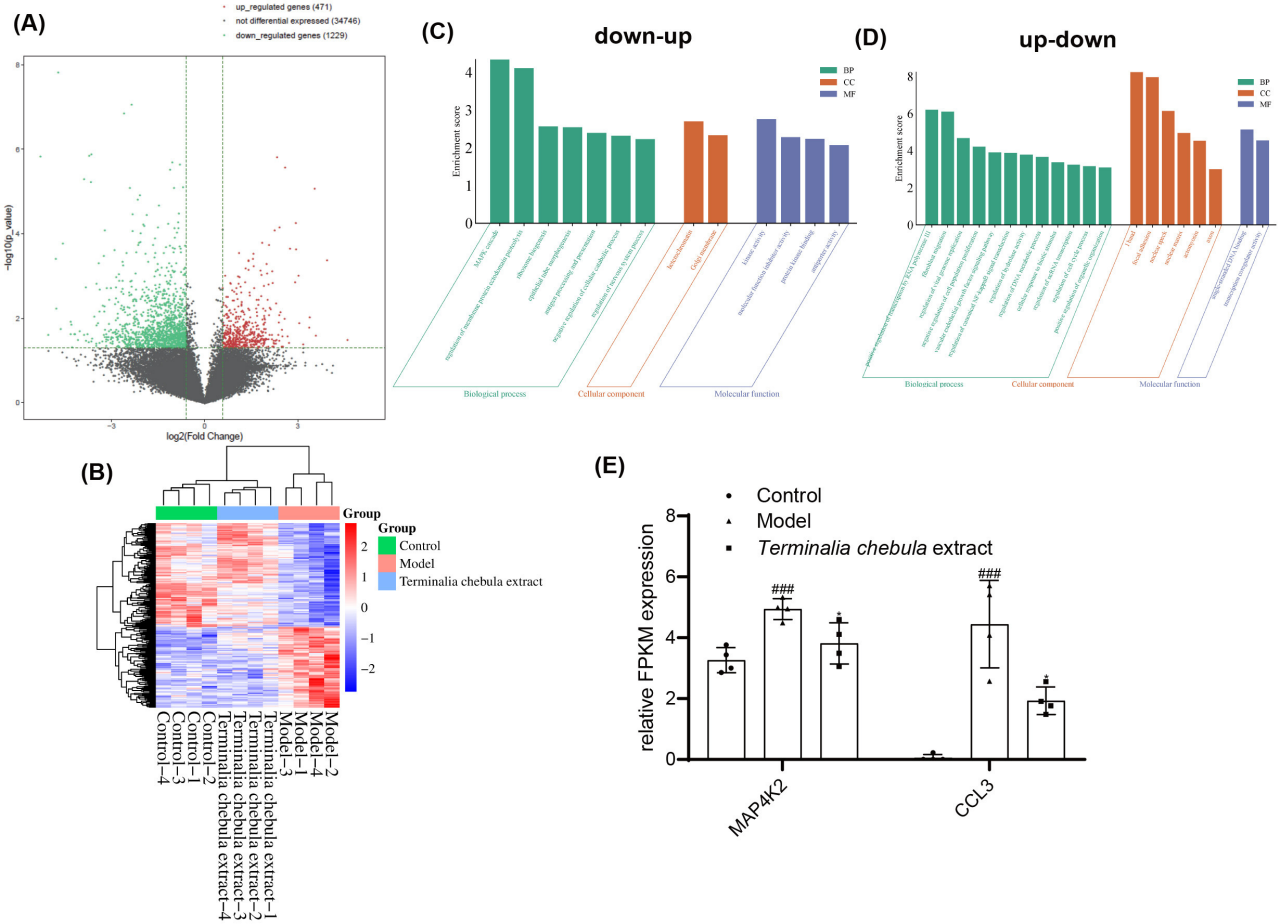
**(A)** Volcano plot comparing the inflammaging group with the *Terminalia chebula* extract group regarding upregulated, non-differentially expressed, and downregulated genes in the transcriptomic profile of inflammatory senescence. **(B)** Cluster analysis of up-down and down-up genes within that transcriptomic profile of inflammatory senescence influenced by *Terminalia chebula* extract [Control: 1640: HSF cells treated with RPMI1640 complete medium; Model: HSF cells treated with M1 THP-1 cell culture medium (inflammaging group); *Terminalia chebula* extract group: HSF cells treated with M1 THP-1 cell culture medium and *Terminalia chebula* extract; Up-down: The gene expression of the inflammaging group was upregulated compared to the control group and downregulated compared to the *Terminalia chebula* extract group; Down-up: The gene expression of the inflammaging group was downregulated compared to the control group and upregulated compared to the *Terminalia chebula* extract group; the color indicates the level of gene expression, with red representing higher expression and blue indicating lower expression]. **(C, D)** GO functional cluster analysis of *Terminalia chebula* extract. Up-down: The gene expression of the inflammaging group was upregulated compared to the control group and downregulated compared to the *Terminalia chebula* extract group; Down-up: The gene expression of the inflammaging group was downregulated compared to the control group and upregulated compared to the *Terminalia chebula* extract group. **(E)** Effect of *Terminalia chebula* extract on genes related to the MAPK cascade.

Similar to RNA-seq in the supramolecular bakuchiol group, the volcano plot indicates that, compared to the inflammaging group, the *Terminalia chebula* extract group previously upregulated 471 genes and downregulated 1,229 genes after sample treatment (Figure 6A). Subsequently, we focused on the genes that are upregulated and downregulated in the supramolecular bakuchiol group; 242 significantly differentially expressed genes were identified in the control group, the inflammaging group, and the *Terminalia chebula* extract group (*p* < 0.05). Compared to the control group, the inflammaging group previously upregulated 93 genes and later downregulated them (up-down) after sample treatment. Meanwhile, 119 genes were initially downregulated in the inflammaging group and subsequently upregulated (down-up) after sample treatment. The cluster heat map illustrates the changes in gene expression levels related to up-down and down-up statuses in these three groups (Figure 6B).

The functions of the up-down and down-up related genes were analyzed using the GO database and annotated with GO terms (Figures 6C, D). The results showed that up-down related genes were mainly enriched in the MAPK cascade and the assembly of cell processes associated with the plasma membrane in biological processes, late endosomes in cellular components, and molecular function inhibitor activity. Down-up genes have a significant impact mainly on biological processes, such as regulating classical nuclear transcription factor-kappa B (NF-κB) signal transduction, the vascular endothelial growth factor (VEGF) signaling pathway, negative regulation of cell population proliferation, positive regulation of transmembrane transport of atoms and ions, cellular response to lipids, and regulation of cell cycle progression, among others.

MAPK cascades have been implicated in various cellular processes, including proliferation, differentiation, apoptosis, cell survival, motility, metabolism, stress response, and inflammation. MAP4K2 acts as a MAP kinase kinase kinase kinase (MAP4K), serving as an upstream activator of the stress-activated protein kinase/c-Jun N-terminal kinase (SAP/JNK) signaling pathway and also activating the p38 MAPKs signaling pathway. According to the sequencing results, *MAP4K2* gene expression is significantly upregulated in senescent HSF cells (Figure 6E). The expression of MAP4K2 can be inhibited following treatment with *Terminalia chebula* extract. CCL3 is a factor with both inflammatory and chemotactic properties. As shown in Figure 5C, CCL3 was significantly reduced after treatment with *Terminalia chebula* extract, indicating that *Terminalia chebula* extract can inhibit inflammaging by reducing cell senescence and the accumulation of inflammatory factors.

## 4 Discussion

Sensitive skin tends to react to oxidative stress and other stimulating factors that can lead to skin inflammation and result in inflammaging, which is a new branch of aging-related research (3, 31). Effective *in vitro* models for sensitive skin are needed to screen anti-inflammaging cosmetic ingredients rapidly, rather than traditional animal models or clinical studies that have been used thus far (14, 15). However, there is a lack of an ingredient screening method specifically designed for inflammaging. To further improve research methods on sensitive skin and determine the anti-inflammaging properties, we established an *in vitro* inflammaging model. In this model, THP-1 cells were induced into an M1 phenotype with upregulated expression of inflammatory factors such as CD86, TNF-α, IL-1β, and IL-6. Next, the supernatant from the M1 THP-1 cell culture was applied to induce aging in HSF cells. Cellular senescence and skin aging indicators p16, p21, and MMP-1 were upregulated, while Col-1 was downregulated, indicating that the *in vitro* cellular model system of inflammaging was successfully constructed.

Additionally, two ingredients, supramolecular bakuchiol and *Terminalia chebula* extract, were utilized to evaluate the feasibility of this system and validate their efficacy against inflammaging. Both supramolecular bakuchiol and *Terminalia chebula* extract were confirmed to reverse inflammaging in this model by downregulating p16, p21, and MMP-1 while upregulating the production of Col-1. Supramolecular bakuchiol comprises the anti-aging component bakuchiol combined with the anti-inflammatory ingredient matrine, which enhances collagen production and inhibits cellular inflammation. Conversely, *Terminalia chebula* extract is reported to have anti-inflammatory properties, allowing it to neutralize inflammatory factors in the M1 supernatant and reduce inflammation in HSF cells.

Moreover, transcriptomics was used to investigate the mechanisms of supramolecular bakuchiol and *Terminalia chebula* extract concerning the reversal of inflammaging. In our study, we found these two ingredients have distinct regulatory mechanisms on inflammaging. Supramolecular bakuchiol enhances collagen production by promoting the gene transcription of *COL1A1* and *COL3A3*, while it inhibits inflammatory factors by increasing the transcription of anti-inflammatory genes [*PTX3* (26, 27), *ADAM33* (28, 29), and *PDLIM1* (30)]. Conversely, *Terminalia chebula* extract can inhibit cell senescence by directly reducing the transcription of *MAP4K2* (32) or by decreasing the accumulation of inflammatory factors, such as CCL3 (33). Our study aligns with previous research indicating that supramolecular bakuchiol primarily exhibits anti-aging properties, while *Terminalia chebula* extract is primarily anti-inflammatory.

Based on this study, we developed a novel *in vitro* model of skin inflammaging using M1 macrophage supernatant to induce HSF cell aging and examined the anti-inflammaging mechanism of *Terminalia chebula* extract and supramolecular bakuchiol with this model. In the future, we aim to enhance the experimental system and broaden the application scope of the inflammaging model to detect not only the direct inflammaging mechanism, primarily between fibroblasts and immune cells but also the indirect inflammaging mechanism, involving keratinocytes that produce ROS to induce aging. Additionally, considering the differences between cell lines and *in vivo* tissues, we will further explore the inflammaging model on a 3D skin model that more closely resembles native tissue and investigate the effects of keratinocytes, fibroblasts, and other common skin cells.

## Data Availability

The data discussed in this publication have been deposited in NCBI's Gene Expression Omnibus (Edgar et al., 2002) and are accessible through GEO Series accession number GSE286316 (https://www.ncbi.nlm.nih.gov/geo/query/acc.cgi?acc=GSE286316).
